# European Domestic Horses Originated in Two Holocene Refugia

**DOI:** 10.1371/journal.pone.0018194

**Published:** 2011-03-30

**Authors:** Vera Warmuth, Anders Eriksson, Mim A. Bower, Javier Cañon, Gus Cothran, Ottmar Distl, Marie-Louise Glowatzki-Mullis, Harriet Hunt, Cristina Luís, Maria do Mar Oom, Isabel Tupac Yupanqui, Tomasz Ząbek, Andrea Manica

**Affiliations:** 1 Department of Zoology, University of Cambridge, Cambridge, United Kingdom; 2 McDonald Institute for Archaeological Research, University of Cambridge, Cambridge, United Kingdom; 3 Departamento de Producción Animal, Facultad de Veterinaria, Universidad Complutense, Madrid, Spain; 4 Animal Genetics Laboratory, VIBS, CVM, Texas A&M University, College Station, Texas, United States of America; 5 Institute for Animal Breeding and Genetics, University of Veterinary Medicine Hannover, Hannover, Germany; 6 Institute of Genetics, Vetsuisse Faculty, University of Bern, Bern, Switzerland; 7 Museus da Politécnica (MNHN & MCUL), Universidade de Lisboa, Lisbon, Portugal; 8 Centro de Biologia Ambiental, Universidade de Lisboa, Lisbon, Portugal; 9 Laboratory of Genomics, National Research Institute of Animal Production, Krakowska 1, Balice, Polan; University of York, United Kingdom

## Abstract

The role of European wild horses in horse domestication is poorly understood. While the fossil record for wild horses in Europe prior to horse domestication is scarce, there have been suggestions that wild populations from various European regions might have contributed to the gene pool of domestic horses. To distinguish between regions where domestic populations are mainly descended from local wild stock and those where horses were largely imported, we investigated patterns of genetic diversity in 24 European horse breeds typed at 12 microsatellite loci. The distribution of high levels of genetic diversity in Europe coincides with the distribution of predominantly open landscapes prior to domestication, as suggested by simulation-based vegetation reconstructions, with breeds from Iberia and the Caspian Sea region having significantly higher genetic diversity than breeds from central Europe and the UK, which were largely forested at the time the first domestic horses appear there. Our results suggest that not only the Eastern steppes, but also the Iberian Peninsula provided refugia for wild horses in the Holocene, and that the genetic contribution of these wild populations to local domestic stock may have been considerable. In contrast, the consistently low levels of diversity in central Europe and the UK suggest that domestic horses in these regions largely derive from horses that were imported from the Eastern refugium, the Iberian refugium, or both.

## Introduction

The domestication of horses was a fundamental step in the history of humankind, providing horse-centred societies with enormous advantages over agricultural societies with regard to long-distance travel, warfare and trade. Consistent with the preference of horses for predominantly open landscapes, the earliest evidence for horse domestication (morphometric data, horse milk residues in pots, and tooth wear resembling that of frequently bitted horses) appears in the Eurasian steppes around 3500 BCE [1], [2]. In a recent study, [3] provide further evidence for the importance of the Eurasian steppe in horse domestication by showing that coat colours other than the wild type first arose in Siberia and Eastern Europe, probably reflecting human selection.

Around the time when the first domesticated horses appeared in the Eurasian steppes, large parts of Europe were still covered by vast expanses of dense forest [4], a habitat that horses avoid [5]. Accordingly, the fossil record for wild horses at that time is extremely scarce [6], [7], suggesting that European domestic horses largely descend from stock that was imported from elsewhere in a process known as demic diffusion [8] (colonisation of an area through population movement [9]). On the other hand, recent mitochondrial DNA (mtDNA) sequence data from a large number of both pre-domestic and domestic horses has shown that European wild populations also contributed to the gene pool of domestic horses [10], [11]. Unfortunately, it is currently difficult to distinguish between regions in Europe where the genetic contribution of local wild horses to domestic stock was substantial, and regions where domestic stock was largely introduced, and backcrossing with local wild horses played only a minor role.

To identify primary areas of horse domestication in Europe, we investigate spatial patterns of genetic diversity in horse breeds for which empirical evidence demonstrates a historic origin in a distinct region of mainland Europe or the UK (henceforth referred to as “traditional breeds”). For the purpose of this paper, we define primary areas of horse domestication as regions where local domestic populations largely descend from local wild stock, be it through their initial recruitment to found domestic populations (“independent” domestication), their extensive introgression into local domestic populations, or both.

If there were only a few, geographically restricted regions in Europe where the genetic contribution of local wild horses to domestic stock was substantial, and if domestic populations from such areas were imported into regions where local wild stock was scarce, we would expect the former areas to have retained high levels of genetic diversity, and the latter areas to be characterised by low levels of diversity. The rationale behind this reasoning is that, as populations expand out of origins, genetic diversity will be lost as a consequence of the (usually) small population sizes involved in such expansions (“founder effect”), see [12] for review). Clear declines in autosomal genetic diversity (allelic richness, heterozygosity) with increasing distance from primary areas of domestication have been found in a number of livestock species, such as cattle [13]–[16], sheep [17], [18], and goats [19].

To investigate spatial patterns of autosomal genetic diversity in European horses, we assembled a unique dataset of more than 1100 horses typed at 12 autosomal microsatellite loci (Table 1), using both new and previously published data. The combined dataset (Table 2) represents the largest and most comprehensive microsatellite dataset on traditional European horse breeds to date.

**Table 1:** Summary of microsatellite markers included in this study.

locus	ECA	primer 5′-3′	reference
AHT4	24	AACCGCCTGAGCAAGGAAGT GCTCCCAGAGAGTTTACCCT	[47]
AHT5	8	ACGGACACATCCCTGCCTGC GCAGGCTAAGGAGGCTCAGC	[47]
HMS3	9	CCAACTCTTTGTCACATAACAAGA GCCATCCTCACTTTTTCACTTTGTT	[48]
HMS6	4	CTCCATCTTGTGAAGTGTAACTCA GAAGCTGCCAGTATTCAACCATTG	[48]
HMS7	1	CAGGAAACTCTCATGTTGATACCATC GTGTTGTTGAAACATACCTTGACTGT	[48]
HTG4	9	CTATCTCAGTCTTGATTGCAGGAC GCTCCCTCCCTCCCTCTGTTCTC	[48]
VHL20	30	CAAGTCCTCTTACTTGAAGACTAG AACTCAGGGAGAATCTTCCTCA	[49]
ASB2	15	CACTAAGTGTCGTTTCAGAAGG GCACAACTGAGTTCTCTGATAGG	[50]
HTG7	4	CCTGAAGCAGAACATCCCTCCTTG ATAAAGTGTCTGGGCAGAGCTGCT	[51]
HMS2	10	CTTGCAGTCGAATGTGTATTAAATG ACGGTGGCAACTGCCAAGGAAG	[48]
HTG10	21	CAATTCCCGCCCCACCCCCGGCA GTTTTTATTCTGATCTGTCACATTT	[51]
HTG6	15	CCTGCTTGGAGGCTGTGATAAGAT GTTCACTGAATGTCAAATTGTGCT	[52]

ECA: location on the horse chromosome.

**Table 2:** Horse breeds included in this study.

breed	ID	origin	N	H	R_S_ (N = 17)	U	F_IS_	reference
Akhal Teke	AT	Turkmenistan	55	0.700	6.09	3	0.069	[33]
Connemara	CO	Ireland (west)	45	0.731	5.73	0	−0.052	[33]
Dales	DL	England (north)	42	0.653	5.11	0	−0.076	[33]
Exmoor	EX	England (southwest)	98	0.611	4.09	1	0.006	[33]
Garrano	GR	Portugal	37	0.763	6.56	0	0.067	[33]
Haflinger	HF	Austria (Tyrol)	45	0.634	4.54	0	0.019	[33]
Lusitano	LU	Portugal	52	0.690	5.57	1	0.022	[33]
Shetland Pony	SP	Scotland (Shetland Islands)	36	0.666	5.22	0	0.000	[33]
Suffolk Punch	SU	England (southeast)	41	0.724	5.49	1	0.084	[33]
Comtois	COM	France (east)	33	0.664	5.16	2	−0.012	[32]
Asturcón	AST	Spain (northwest)	119	0.733	5.79	1	−0.009	[23]
Jaca Navarra	JNA	Spain (north)	122	0.729	5.98	3	0.035	[23]
Losino	LOS	Spain (north)	66	0.704	5.79	0	−0.022	[23]
Caballo Gallego	PGL	Spain (northwest)	72	0.762	6.82	4	0.060	[23]
Pottoka	POT	Spain (north)	51	0.775	6.52	0	0.043	[23]
Altmark Draught	AMD	Germany (east)	31	0.647	4.89	0	−0.009	New data
Caspian Horse	CAS	Iran	30	0.770	6.70	2	−0.002	New data
Camargue	CMG	France (south)	22	0.776	6.43	1	0.111	New data
Highland Pony	HIG	Scotland	25	0.687	5.03	0	−0.033	New data
Hucul	HUP	Carpathian Mountains	17	0.694	5.55	0	0.103	New data
Posavina	POS	Croatia	24	0.695	5.58	0	−0.062	New data
Schleswig Draught	SDH	Germany (north)	22	0.693	4.59	1	−0.041	New data
Noriker	NOS	Austria	26	0.652	5.08	0	0.036	New data
Bilgoraj	BLG	Poland	28	0.724	5.19	na	−0.015	[31]

*N*  =  sample size, *H*  =  Nei's gene diversity, *R*_S_  =  allelic richness, *U*  =  number of private alleles, *F*_IS_ =  inbreeding coefficient, na  =  not determined because dataset could not be aligned with the rest due to lack of reference samples.

## Results

### Spatial patterns of genetic diversity in traditional European horse breeds

**Figure 1:**
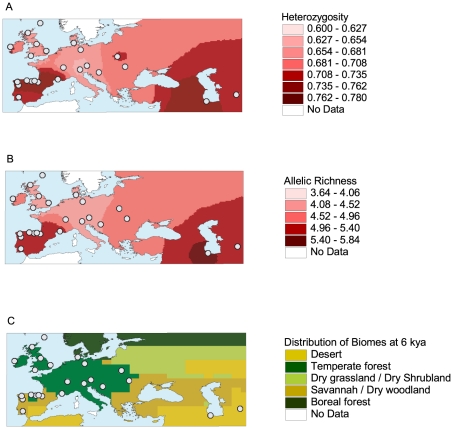
High diversity in European horses mirrors the distribution of open landscape in the mid-Holocene. (A) Interpolation of expected heterozygosity *H* in 24 old European horse breeds. High levels of genetic diversity, as indicated by dark shading, are found in the Caspian region of western Asia and the Iberian Peninsula. White circles indicate the approximate location of origin for each breed. (B) Interpolation of allelic richness *R*_S_ in 24 native European breeds using a minimum sample size of N = 17. (C) Spatial distribution of biomes in Europe and western Asia 6000 years ago (6 ka) as inferred from model simulations. [Map adapted from 19].

Geographic variation in gene diversity (*H*) reveals two hotspots of diversity, one in the Caspian region of western Asia, our easternmost sampling location, and one in the Iberian Peninsula (Fig. 1A). A very similar pattern is obtained for allelic richness (*R_S_*, Fig. 1B). The Iberian hotspot coincides with the only region in central and western Europe that was characterised by appreciable expanses of open landscape in the mid-Holocene (Fig.1C, adapted from [20]), suggesting that not only the Eurasian steppes but also the Iberian Peninsula served as refugia for wild horses in the early and mid Holocene, when vast expanses of forest would have rendered most of Europe unsuitable for this steppe-adapted species.

**Figure 2:**
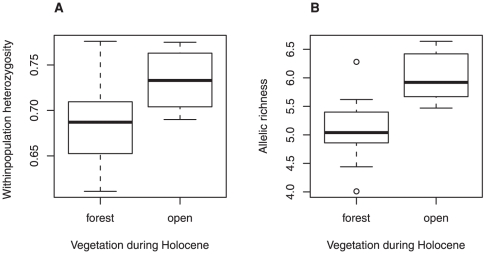
Levels of genetic diversity in Iberia (group: “open”) and central Europe/Britain (group: “forested”). (A) Average gene diversity *H* and (B) average allelic richness *R*_S_ per group (“open”: N = 9; “forested”: N = 15). Statistical significance was determined using a two-sided permutation test (* p<0.05) and 10,000 randomisations.

In a comparison of diversity between breeds from regions that were predominantly open versus those that were predominantly forested at 6 ka, we find that the latter group has significantly lower diversity (median *H* = 0.687, median *R*_S_ = 4.42) than the former (median *H* = 0.733, median *R*_S_ = 5.09; two-sided permutation tests with 10,000 runs; gene diversity *H*: p = 0.006, Fig. 2A; allelic richness *R*_S_ *:* p = 0.002, Fig. 2B). Low levels of diversity in breeds from previously forested areas are consistent with a loss of diversity as small herds of domestic horses were imported into these areas, following their domestication in Iberia or the Eastern steppes. Estimating the relative contribution of the two refugial populations to individual breeds is not possible here due to the limited number of markers used.

### Ancient history or recent demography

**Figure 3:**
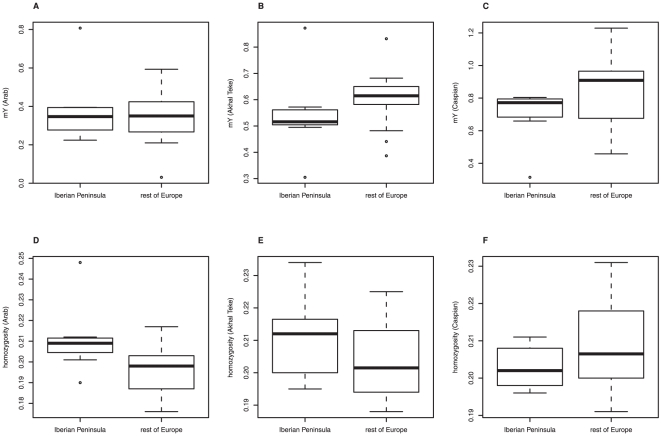
Levels of admixture from three Middle Eastern breeds in Iberia and central Europe/Britain. (A–C) Relative genetic contribution of the (A) Arab, (B) Akhal Teke, and (C) Caspian breed to Iberian and cE/UK breeds based on the admixture coefficient *m*_Y_. (D–F) Relative genetic contribution of the (D) Arab, (E) Akhal Teke, and (F) Caspian breed to Iberian and cE/UK breeds based on expected homozygosity *F*_S_.

The observed genetic pattern could be a consequence of recent demographic processes: high diversity in Iberia might reflect disproportionally high levels of admixture from high-diversity non-Iberian breeds. Similarly, low diversity in central Europe and the UK (cE/UK) might reflect disproportionally severe recent bottlenecks or higher levels of inbreeding in breeds from these areas. Since domestication, horses from the Middle East have been among the most widely used to “improve” horse breeds across Europe [21]. We estimated the genetic component of three Middle Eastern breeds (Arab, Akhal Teke and Caspian) in Iberian and cE/UK breeds using two different measures of admixture, the admixture coefficient m_Y_ (Table S1A–C) and expected homozygosity *F*_S_ (Table S2). We found no significant difference in the level of admixture from Middle Eastern breeds between Iberian and cE/UK horses (Wilcoxon tests, admixture with Arab: *m*_Y_: W = 43, p = 0.877; *F*_S_: W = 72.5, p = 0.086; admixture with Akhal Teke: *m*_Y_: W = 28, p = 0.183; *F*_S_: W = 63.5, p = 0.296; admixture with Caspian: *m*_Y_: W = 23, p = 0.081; *F*_S_: W = 37, p = 0.389; Fig. 3A–E). Similarly, there is no significant difference in *F*_IS_ between Iberian and cE/UK breeds (Wilcoxon test, W = 70.5, p = 0.217; median (IQR) Iberia: 0.035 (0.007–0.052); cE/UK: −0.009 (−0.037–0.028)), implying that breeding practices are unlikely to explain the observed pattern in diversity. Furthermore, cE/UK breeds, but not Iberian ones, would have had to undergo extreme recent contraction, to average effective population sizes (*N*_e_) of between ten and 20 individuals, to generate the observed pattern (equation (3)). While a few individual breeds are known to have undergone such severe bottlenecks in the recent past, these include breeds from the proposed refugia [22]–[24]. Based on the evidence presented here, we infer that the observed pattern of genetic diversity is unlikely to be the result of recent demographic processes.

## Discussion

Our investigation of genetic diversity in traditional European horse breeds reveals two hotspots of genetic diversity, one in the Caspian region of western Asia and one in the Iberian Peninsula. The distribution of high genetic diversity in European horses coincides with the distribution of open vegetation in the mid-Holocene, suggesting that these areas acted as refugia for wild horses at a time when most of Europe was covered by dense forest [25].

Although our lack of sampling locations in Central and East Asia prevents us from pinpointing primary areas of horse domestication in this region, high diversity in the Caspian area is in agreement with palaeontological data suggesting that *E. ferus* survived the Holocene in South-West Asia and Central Asia [26]. Additional sampling further east will help establish whether there is a genuine hotspot of genetic diversity in the Caspian region or whether high diversity in this region merely reflects generally higher levels of diversity in the Eurasian steppes.

A hotspot of genetic diversity in the Iberian Peninsula indicates that *E. ferus* may have also survived in Iberia. The Iberian Peninsula was the only region in central and western Europe in which appreciable expanses of open habitat persisted throughout the Holocene [27], [28]. The presence of wild horses in the Iberian Peninsula prior to domestication is supported by findings of horse remains in Neolithic and Copper Age sites (sixth to fourth millennium B.P., [29], [30]). More recently, it has been shown that several pre-domestic Iberian maternal lineages survive in modern horses of Iberian descent [10], [11], thus documenting a genetic contribution of Iberian wild stock to local domestic horses. Here we go on to show that the genetic contribution of Iberian wild stock to local domestic horses may have been substantial: the high diversity in Iberian horses is consistent with the persistence of *E. ferus* in the Iberian Peninsula from the Pleistocene through the Holocene, and the subsequent extensive use of local Iberian wild horses in establishing and/or restocking local domestic populations.

Hypotheses of local domestication in other parts of Europe could not be confirmed in this study. Levels of genetic diversity in breeds from previously forested areas are consistently low, suggesting a scenario whereby these areas primarily relied on an import of horses from either the Iberian or the Asian or both refugia (i.e. demic diffusion). This is consistent with the fossil record for horses, which, in turn, reflects the ecology of this large, group-living animal. While our results do not imply that wild horses were entirely absent from forested parts of Holocene Europe, we suggest that their presence in these regions was spatially and temporally discontinuous, with local extinctions and re-colonisations occurring in response to natural forest gap dynamics.

In this study, we have confirmed previous claims whereby populations of *E. ferus* persisted in refugial steppe habitat in the East [26], and provide further evidence for a second Holocene refugium for wild horses in the Iberian Peninsula. Our results suggest that primary areas of horse domestication were confined to regions where considerable expanses of open landscape persisted throughout the Holocene, and that previously forested regions in Europe primarily relied on an import of domestic horses. Whether the knowledge of how to successfully capture, tame and breed horses reached Iberia through cultural transmission, or whether this knowledge was acquired independently, is an open question that cannot be answered with genetic data. The approach used here will provide further insights into the processes involved in horse domestication when applied to the Eurasian steppes, a region which has been shown to have played a central role in horse domestication.

## Methods

### Datasets

In this paper, we present new genotyping data supplemented by microsatellite data from four published studies [23], [31]–[33]. Individual datasets were aligned using a minimum of four reference samples from each participating lab. The dataset from [32] had been standardised to reference samples from the ISAG Horse Comparison Test and could therefore be aligned directly. Due to the lack of reference samples, the dataset of [31] could not be aligned with the rest. The breed contained in this dataset (Bilgoraj) was therefore only used in comparisons of within-population diversity.

### Choice of samples

For our final dataset, we excluded all non-European breeds as well as breeds that are known to have been introduced to various European islands in recent times. In order to maximise our chances to detect signals of domestication, we furthermore excluded modern “warmblood” breeds which, by definition, are composite breeds with varying contributions of “heavy” draft horses and “light” riding horses [21]. Our a-priori rules for the inclusion of breeds therefore focused on pony and draft horse breeds for which a historic founding date can be demonstrated, including breeds which are known to have been crossbred with Middle Eastern breeds and/or the English Thoroughbred. Our final dataset includes 1167 individuals from 24 traditional breeds from mainland Europe and the UK (Table 2).

### DNA extraction and PCR amplification

#### Previously unpublished data

DNA was extracted from blood or hair. For DNA extraction from blood see the Methods section in [34]. DNA extraction from hair was carried out according to a protocol adapted from [35] using 15–20 hair roots per individual. DNA extracts were purified (QIAquick purification kit, Qiagen) and standardised to a concentration of 10 ng of DNA/µl. A total of 12 microsatellite loci (Table 1) was amplified in multiplex polymerase chain reactions (PCRs) adapted from [32]. PCR amplifications were carried out in a total volume of 12.5 µl using a microsatellite genotyping kit (Qiagen) with 10 ng template DNA and 1.25 µl of a 1∶10 dilution of primer mix. PCR reactions were performed on a thermal cycler under the following cycling conditions: 95°C for 6 min; 32 cycles of 95°C for 30 sec, 58°C for 90 sec, 72°C for 30 sec; 60°C for 30 min. PCR products were run on an ABI 3730 Genetic Analyser (Applied Biosystems). Alleles were assigned using GeneMapper Software v37 (Applied Biosystems).

#### Published datasets

For DNA extraction and PCR amplification protocols of the previously published datasets, please refer to the original publications [23], [31]–[33].

### Data analysis

#### Genetic Diversity

Nei's gene diversity *H* [36] and the inbreeding coefficient *F*_IS_ [37] were estimated using FSTAT v 2.9.3.2 [38]. Allelic richness was estimated using the rarefaction algorithm implemented in the programme ADZE [39]. The estimates of allelic richness were standardised to the smallest sample size in our dataset, *N* = 17. Private alleles were determined using GDA [40]. Permutation tests were carried out in FSTAT and Wilcoxon tests were carried out in R [41].

#### Spatial interpolation of genetic diversity

Because of the uneven sampling of populations across Europe, we developed an approach based on Gaussian kernel interpolation that allows for an adaptive kernel width. Using a hexagonal grid representation of Eurasia (grid points spaced approximately 110 km apart, each land grid point is connected to up to six neighbours as in [42], [43]) we calculated the shortest distance 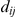 on land from each grid point *i* to each sample location *j*. United Kingdom, Ireland, and Shetland were connected to the rest of the graph by creating suitable “landbridges”. For each grid point *i* we then estimated the value of genetic diversity (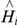) using Gaussian kernel interpolation,



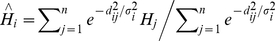
(1)where *n* is the number of sample locations, *H_j_* is the genetic diversity for location *j*, and 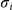 is the kernel width for the grid point *i*. Because sample points are clustered, with dense sampling in western Europe and very sparse sampling in the East, we chose the width 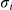 of the kernel in grid point *i* to be proportional to the harmonic average of the distance to the sample locations (in order to avoid artefacts from the finite resolution of the grid, distances are forced to be at least 100 km, the typical distance between neighbouring grid points):
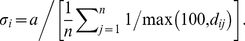
(2)The scale factor *a* was chosen to *a* = 23, such that the kernel 
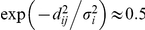
when the distance 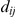 is twice the distance to the closest sample point. Finally, we used Arcview v32 (ESRI) to produce the figures from the grid point estimates.

### Potential confounding effects from recent demography

The observed genetic pattern could be a consequence of recent demographic processes rather than a signal of domestication. We considered three major confounding factors that would invalidate the interpretation of genetic hotspots as centres of origin: admixture, recent population declines, and population substructure.

#### Admixture

In the recent past, breeds from the Middle East have been widely used to “improve” horse breeds throughout Europe [21]. Since admixture can affect patterns of genetic diversity, we estimated the contribution of three Middle Eastern horse breeds (Arab, Akhal Teke, Caspian) to all other breeds in our dataset. We used two measures of admixture: the admixture coefficient *m*_Y_ [44] and expected homozygosity *F*_S_. *m*_Y_ coefficients and standard deviations were computed as averages of 1,000 random bootstrap samples using the programme ADMIX [44], [45]. The calculation of *m*_Y_ is based on the assumption that allele frequencies in the admixed populations are linear combinations of those in the parental populations; contrary to other admixture coefficients, *m*_Y_ takes into account allele frequency differences as well as the degree of molecular divergence between alleles and has been shown to be appropriate for use with microsatellite data [44]. Since the true parental populations (i.e. European populations of wild horses) are not available, we chose the Hucul, a breed which has been bred in the Carpathian Basin since the thirteenth century [21], to represent the genetic component of non-Middle Eastern breeds. The relative genetic contribution of the Middle Eastern breeds to central European/UK breeds was established by individual comparison of each of the three Middle Eastern breeds with the Hucul breed. Since the surrogate parental populations chosen here are unlikely to represent the genetic variability present in the true parental populations, the resulting *m*_Y_ values merely describe the *relative* contribution of the surrogate parental populations to the admixed populations, not their absolute contributions.

#### Effect of population substructure on within-population heterozygosity

If mating is non-random, substructure within breeds may arise, causing a reduction in overall heterozygosity (Wahlund effect). This reduction can be measured using *F*_IS_ [37]. If the decreased diversity in central Europe/the UK arose because breeding practices in this area have promoted stronger population substructure than those in the proposed refugia, we would expect to see a higher proportion of positive *F*_IS_ values in the former.

#### Recent declines in population sizes

Recent bottlenecks might have contributed to the low diversity observed in central Europe and Great Britain (cE/UK), as compared to Iberia and western Asia. We explored the magnitude of the bottlenecks that would have been necessary to produce the lower median diversity found in cE/UK using the recursion

*H_t+_*_1_ = H_t_*(1-1/2*N_t_*), (3)where *H_t_* is the within-population heterozygosity and *N*_t_ the effective population size in generation t. We set the initial diversity *H*_t_ equal to the median diversity observed in the putative refugial populations. This is a very conservative estimate, since it (incorrectly) assumes that the latter did not experience recent declines in population sizes.

We considered scenarios in which central European and British populations were reduced to minimum effective population sizes of *N* = 10, 20, 30, 40, or 50 either six or three generations ago, and then recovered at an annual growth rate *r* equal to 1.1. Using a generation time of 12 years, the bottlenecks coincide with the 1940s and the 1970s, two periods in which many native horse breeds in Europe experienced dramatic declines in population sizes [46].

## Supporting Information

Table S1 **Admixture coefficients** ***m*** **_Y_ for all breeds using (A) Arab and Hucul, (B) Akhal Teke and Hucul, and (C) Caspian and Hucul as parental populations.**
(PDF)

Table S2 **Expected homozygosity (** ***F_S_*** **) of different European horse breeds with the Arab, Akhal Teke, and Caspian, respectively.**
(PDF)
