# Correction: European Domestic Horses Originated in Two Holocene Refugia

**DOI:** 10.1371/annotation/ef679268-70cb-49f8-8e1b-2c9df4ed1930

**Published:** 2011-05-06

**Authors:** Vera Warmuth, Anders Eriksson, Mim A. Bower, Javier Cañon, Gus Cothran, Ottmar Distl, Marie-Louise Glowatzki-Mullis, Harriet Hunt, Cristina Luís, Maria do Mar Oom, Isabel Tupac Yupanqui, Tomasz Ząbek, Andrea Manica

There is information missing from the funding section. The complete funding information is: 

"This work was partially supported by a research studentship from the Biotechnology and Biological Sciences Research Council(BB/E527604/1) and a PhD studentship from the German Academic Exchange Service (D/07/44562) to VW, a Leverhulme Trust project grant (F/09 757/B) to MAB, a Philip Leverhulme Award and a Biotechnology and Biological Sciences Research Council grant (BB/H005854/1) to AM. The funders had no role in study design, data collection and analysis, decision to publish, or preparation of the manuscript."

