# Supplementary material for: European Domestic Horses Originated in Two Holocene Refugia
Source: PLoS One. 2011 Mar 30;6(3):e18194. doi: 10.1371/journal.pone.0018194 (PMC3068172; doi:10.1371/journal.pone.0018194)
Supplement: Table S1 — Admixture coefficients m Y for all breeds using (A) Arab and Hucul, (B) Akhal Teke and Hucul, and (C) Caspian and Hucul as parental populations. (PDF) [file pone.0018194.s001.pdf]

Supplementary Table S1. (A) Admixture coefficients m_Y for all breeds using Arab and Hucul as parental populations.

		Arab		Hucul	
**Breed**	**Group**	**Bootstrap* average**	**Bootstrap standard deviation**	**Bootstrap average**	**Bootstrap standard deviation**
Altmark Draught	Central Europe/Britain	0.4006	0.1795	0.5994	0.1795
Camargue	Central Europe/Britain	0.4849	0.0942	0.5151	0.0942
Comtois	Central Europe/Britain	0.3497	0.1845	0.6503	0.1845
Connemara	Central Europe/Britain	0.4237	0.1306	0.5763	0.1306
Dale	Central Europe/Britain	0.2307	0.1235	0.7693	0.1235
Exmoor	Central Europe/Britain	0.3232	0.1178	0.6768	0.1178
Haflinger	Central Europe/Britain	0.3242	0.1693	0.6758	0.1693
Highland	Central Europe/Britain	0.5179	0.1105	0.4821	0.1105
Noriker	Central Europe/Britain	0.2098	0.1549	0.7902	0.1549
Posavina	Central Europe/Britain	0.2673	0.1314	0.7327	0.1314
Schleswig Draught	Central Europe/Britain	0.3980	0.1278	0.6020	0.1278
Shetland	Central Europe/Britain	0.0311	0.1389	0.9689	0.1389
Suffolk Punch	Central Europe/Britain	0.5926	0.0857	0.4074	0.0857
Asturcon	Iberian Peninsula	0.2824	0.1199	0.7176	0.1199
Caballo Gallego	Iberian Peninsula	0.3472	0.0900	0.6528	0.0900
Garrano	Iberian Peninsula	0.3934	0.0987	0.6066	0.0987
Jaca Navarra	Iberian Peninsula	0.3947	0.1050	0.6053	0.1050
Losino	Iberian Peninsula	0.2244	0.0969	0.7756	0.0969
Lusitano	Iberian Peninsula	0.8069	0.0934	0.1931	0.0934
Pottoka	Iberian Peninsula	0.2722	0.0865	0.7278	0.0865

* m_Y estimates and standard deviations were determined by averaging over 1,000 random bootstrap samples

Supplementary Table S1. (B) Admixture coefficients m_Y for all breeds using Akhal Teke and Hucul as parental populations.

Breed	group	Akhal Teke		Hucul	
		Bootstrap* average	Bootstrap standard deviation	Bootstrap average	Bootstrap standard deviation
Altmark Draught	Central Europe/Britain	0.6061	0.1329	0.3939	0.1329
Camargue	Central Europe/Britain	0.6185	0.0927	0.3815	0.0927
Comtois	Central Europe/Britain	0.5890	0.1440	0.4110	0.1440
Connemara	Central Europe/Britain	0.6154	0.1030	0.3846	0.1030
Dale	Central Europe/Britain	0.6303	0.0864	0.3697	0.0864
Exmoor	Central Europe/Britain	0.4822	0.0934	0.5178	0.0934
Haflinger	Central Europe/Britain	0.6499	0.1156	0.3501	0.1156
Highland	Central Europe/Britain	0.5825	0.1038	0.4175	0.1038
Noriker	Central Europe/Britain	0.6658	0.1055	0.3342	0.1055
Posavina	Central Europe/Britain	0.4409	0.1110	0.5591	0.1110
Schleswig Draught	Central Europe/Britain	0.6823	0.1033	0.3177	0.1033
Shetland	Central Europe/Britain	0.3874	0.0947	0.6126	0.0947
Suffolk Punch	Central Europe/Britain	0.8321	0.0756	0.1679	0.0756
Asturcon	IberianPeninsula	0.4954	0.0935	0.5046	0.0935
Gallego	IberianPeninsula	0.5160	0.0710	0.4840	0.0710
Garrano	IberianPeninsula	0.5721	0.0854	0.4279	0.0854
JacaNavarra	IberianPeninsula	0.5511	0.0853	0.4489	0.0853
Losino	IberianPeninsula	0.3049	0.0904	0.6951	0.0904
Lusitano	IberianPeninsula	0.8725	0.0775	0.1275	0.0775
Pottoka	IberianPeninsula	0.5140	0.0801	0.4860	0.0801

* m_Y estimates and standard deviations were determined by averaging over 1,000 random bootstrap samples

Supplementary Table S1 (C). Admixture coefficients m_Y for all breeds using Caspian and Hucul as parental populations.

		Caspian		Hucul	
**Breed**	**Group**	**Bootstrap* average**	**Bootstrap standard deviation**	**Bootstrap average**	**Bootstrap standard deviation**
Altmark Draught	Central Europe/Britain	1.1340	0.2697	-0.1340	0.2697
Camargue	Central Europe/Britain	0.6755	0.1745	0.3245	0.1745
Comtois	Central Europe/Britain	0.9095	0.3237	0.0905	0.3237
Connemara	Central Europe/Britain	0.5169	0.3521	0.4831	0.3521
Dale	Central Europe/Britain	0.8336	0.1571	0.1664	0.1571
Exmoor	Central Europe/Britain	0.6042	0.2289	0.3958	0.2289
Haflinger	Central Europe/Britain	1.2294	0.2151	-0.2294	0.2151
Highland	Central Europe/Britain	0.9483	0.9483	0.0517	0.1865
Noriker	Central Europe/Britain	0.9568	0.2146	0.0432	0.2146
Posavina	Central Europe/Britain	0.9654	0.1592	0.0346	0.1592
Schleswig Draught	Central Europe/Britain	0.8452	0.2011	0.1548	0.2011
Shetland	Central Europe/Britain	0.4581	0.2047	0.5419	0.2047
Suffolk Punch	Central Europe/Britain	1.0487	0.1838	-0.0487	0.1838
Asturcon	IberianPeninsula	0.7073	0.2201	0.2927	0.2201
Gallego	IberianPeninsula	0.8043	0.1135	0.1957	0.1135
Garrano	IberianPeninsula	0.7874	0.1425	0.2126	0.1425
JacaNavarra	IberianPeninsula	0.7716	0.1774	0.2284	0.1774
Losino	IberianPeninsula	0.3138	0.2069	0.6862	0.2069
Lusitano	IberianPeninsula	0.8019	0.3376	0.1981	0.3376
Pottoka	IberianPeninsula	0.6589	0.1213	0.3411	0.1213

* m_Y estimates and standard deviations were determined by averaging over 1,000 random bootstrap samples
