# Supplementary material for: European Domestic Horses Originated in Two Holocene Refugia
Source: PLoS One. 2011 Mar 30;6(3):e18194. doi: 10.1371/journal.pone.0018194 (PMC3068172; doi:10.1371/journal.pone.0018194)
Supplement: Table S2 — Expected homozygosity ( FS ) of different European horse breeds with the Arab, Akhal Teke, and Caspian, respectively. (PDF) [file pone.0018194.s002.pdf]

Supplementary Table S2. Expected homozygosity (F_S) of different European horse breeds with the Arab, Akhal Teke, and Caspian, respectively.

**Breed**	**Group**	**Expected homozygosity F_S**		
		**Arab**	**AkhalTeke**	**Caspian**
AltmarkDraught	Central Europe/Britain	0.18	0.20	0.21
Camargue	Central Europe/Britain	0.22	0.22	0.21
Comtois	Central Europe/Britain	0.20	0.19	0.19
Connemara	Central Europe/Britain	0.21	0.22	0.20
Dale	Central Europe/Britain	0.19	0.19	0.21
Exmoor	Central Europe/Britain	0.19	0.21	0.21
Haflinger	Central Europe/Britain	0.18	0.19	0.22
Highland	Central Europe/Britain	0.20	0.21	0.21
Hucul	Central Europe/Britain	0.20	0.19	0.22
Noriker	Central Europe/Britain	0.18	0.20	0.22
Posavina	Central Europe/Britain	0.19	0.20	0.22
SchleswigDraught	Central Europe/Britain	0.20	0.20	0.20
Shetland	Central Europe/Britain	0.21	0.23	0.23
SuffolkPunch	Central Europe/Britain	0.19	0.21	0.20
Asturcon	IberianPeninsula	0.20	0.20	0.21
Gallego	IberianPeninsula	0.21	0.21	0.20
Garrano	IberianPeninsula	0.21	0.21	0.20
JacaNavarra	IberianPeninsula	0.21	0.22	0.21
Losino	IberianPeninsula	0.21	0.20	0.20
Lusitano	IberianPeninsula	0.25	0.23	0.21
Pottoka	IberianPeninsula	0.19	0.20	0.20
